# Functional motif detection via *in silico* ablation using AlphaGenome

**DOI:** 10.1093/bib/bbag422

**Published:** 2026-08-06

**Authors:** Yuxuan Liang, Sebastian A Dziadowicz, Lei Wang, Gangqing Hu, Pingkun Yan, Ge Wang

**Affiliations:** Department of Biomedical Engineering, Rensselaer Polytechnic Institute, 110 Eighth Street, Troy, NY 12180, United States; Department of Microbiology, Immunology and Cell Biology, West Virginia University, 64 Medical Center Drive, Morgantown, WV 26506-9177, United States; Department of Microbiology, Immunology and Cell Biology, West Virginia University, 64 Medical Center Drive, Morgantown, WV 26506-9177, United States; Department of Microbiology, Immunology and Cell Biology, West Virginia University, 64 Medical Center Drive, Morgantown, WV 26506-9177, United States; Department of Biomedical Engineering, Rensselaer Polytechnic Institute, 110 Eighth Street, Troy, NY 12180, United States; Department of Biomedical Engineering, Rensselaer Polytechnic Institute, 110 Eighth Street, Troy, NY 12180, United States

**Keywords:** functional motif detection, in silico perturbation, transcription factors, chromatin modeling, AlphaGenome

## Abstract

Deciphering which transcription factor (TF) motif instances are functionally required for enhancer activity typically demands ChIP-based assays or labor-intensive perturbation experiments. We introduce a virtual motif-perturbation framework that uses AlphaGenome, a large sequence-to-function foundation model, to infer motif-level regulatory contribution directly from DNA sequence. Candidate C/EBP$\mathrm{\beta} $ motifs are identified within chromatin-active regions and systematically ablated *in silico*; the resulting changes in predicted regulatory activity are quantified and assessed against a sham-derived null distribution to establish statistical confidence. To evaluate whether sequence-level perturbations recapitulate biologically meaningful regulatory dependence, we compared *in silico* predictions with CUT&RUN measurements of H3K27ac following CEBPB knockout in multiple myeloma cells. Key activating motifs exhibited concordant loss of H3K27ac across both settings, whereas loci with apparent discrepancies reflected biologically interpretable mechanisms. Together, these findings suggest that large sequence-based models can approximate the directional consequences of TF perturbation, supporting motif-level functional analysis directly from DNA sequence. This supports sequence-only virtual ablation as a scalable framework for hypothesis generation and regulatory annotation that can be extended to additional TFs, cell types, and chromatin modalities.

## Graphical Abstract

**Graphical Abstract:**
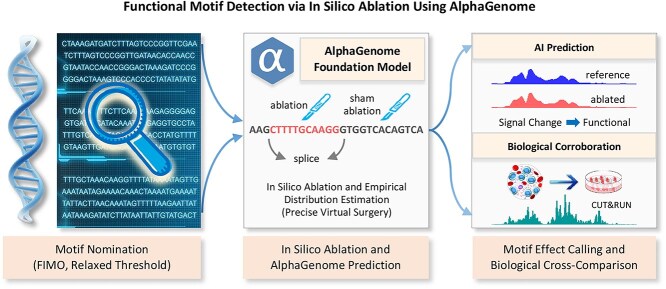

## Introduction

Transcription factors (TFs) regulate gene expression by recognizing short DNA motifs, with individual motif instances serving as fundamental cis-regulatory units whose subtle sequence changes can create, disrupt, or fine-tune TF binding. Identifying which motif occurrences are functionally required for enhancer activity is therefore critical for understanding cell-type–specific regulation, interpreting disease-associated noncoding variants, and revealing regulatory network logic. Although experimental assays such as ChIP-seq remain the standard for mapping TF occupancy [1, 2], their reliance on high-quality antibodies, substantial input material, and extensive optimization limits scalability. Consequently, comprehensive TF-binding maps exist for only a small subset of TFs, and systematic profiling across diverse conditions remains challenging [3], motivating computational strategies to identify functional motif instances without large-scale laboratory assays.

Computational approaches for motif discovery have historically fallen into two categories: statistical frameworks based on sequence-derived motif models and deep learning predictors trained on ChIP-seq or chromatin accessibility data. Classical methods such as position weight matrices (PWMs), k-mer–based models [4, 5], and TRANSFAC-related transcription factor binding site (TFBS) search approaches rely on existing motif representations to scan genomic regions for candidate matches. TRANSFAC provides curated TF information, experimentally supported binding sites, and DNA-binding profiles, and related tools such as Match use weight-matrix libraries to identify putative TFBSs [6, 7]. These approaches are valuable for motif nomination when the relevant motif model is available. However, their outputs are primarily sequence-match scores whose interpretation depends on motif database coverage, background assumptions, and manually selected thresholds. Permissive cutoffs increase false positives by capturing weak or spurious matches, whereas stringent cutoffs risk missing low-affinity but potentially important sites. Moreover, motif matching alone does not estimate how perturbing a specific motif instance would alter local regulatory readouts.

Deep learning models have addressed some limitations of traditional motif analysis by learning regulatory sequence features directly from DNA. Early convolutional architectures such as DeepBind [8] and DeepSEA [9] showed that neural networks can infer TF binding and chromatin accessibility without explicit PWMs. Later models expanded both receptive fields and predictive resolution: BPNet [10] introduced base-resolution profile prediction and uncovered motif syntax such as spacing and cooperativity, while Basenji2 [11] and Enformer [12] extended sequence contexts to hundreds of kilobases and captured long-range enhancer–promoter interactions. Despite these advances, binding-label–trained models remain constrained by the availability and condition specificity of ChIP-seq or DAP-seq data, and their generalization across cell types or species can be incomplete [13].

A central limitation of binding-based labels is their inability to distinguish occupancy from functional relevance. A motif may be bound yet nonfunctional, or may contribute to regulation only under specific chromatin or signaling conditions. Chromatin assays such as ChIP-seq [14], ATAC-seq [15], and DNase-seq [16, 17] identify active regulatory regions, but they cannot determine which individual motif instances are necessary for enhancer output. Experimental perturbation, achieved by mutating or excising candidate motifs and then measuring chromatin or transcriptional responses, provides direct evidence of functional necessity [18, 19]. However, these assays require substantial laboratory throughput and are feasible only for a limited number of loci.

Large sequence-to-function models provide a promising alternative. AlphaGenome [20], trained on >7000 human and mouse functional genomic tracks, jointly learns short-range motif syntax and long-range regulatory interactions across 1 Mb windows. Its sensitivity to small sequence edits indicates that *in silico* motif ablation, implemented by computationally deleting candidate motifs and quantifying the resulting changes in predicted chromatin activity, can approximate experimental perturbation at minimal cost. Because AlphaGenome generates diverse chromatin outputs for the cell types included in its training data, it enables motif-function assessment in those contexts without requiring TF-specific ChIP-seq and therefore shifts motif analysis from predicting occupancy to estimating functional consequence. More broadly, recent sequence-to-function models (including, but not limited to, AlphaGenome) have motivated extensive *in silico* perturbation and attribution analyses (e.g. saturation mutagenesis and virtual edits [21, 22]) to estimate the regulatory impact of noncoding variants and motif changes.

Here we introduce an AlphaGenome-guided framework for computationally inferring functional TF motif instances directly from genomic sequence. Each candidate motif is excised within a 1 Mb context window and the resulting change in predicted regulatory activity is quantified. A pronounced local decrease indicates an activating contribution; an increase suggests a repressive role; and negligible change indicates non-essentiality in that chromatin context. Unlike supervised deep learning methods trained on ChIP-seq or DAP-seq labels, this approach infers motif-level regulatory contribution through the perturbation response and does not require newly generated TF-specific ChIP-seq, TF-specific antibodies, or matched perturbation datasets for the queried loci. This property allows the framework to identify functional motif instances beyond those prioritized by standard PWM-thresholding strategies, including weak or context-dependent sites that may be underrepresented by conventional motif catalogs. Compared with prior variant-centric *in silico* perturbation studies, we treat each motif instance as the unit of perturbation and couple effect calling with a matched sham-deletion null distribution to assign statistical confidence.

To illustrate the utility of this framework, we analyze the chromatin-activity signal H3K27ac and examine a cluster of C/EBP$\mathrm{\beta} $ motifs near the *TSC22D3* locus in multiple myeloma. In MM.1S cells, *in silico* motif ablation uncovers distinct motif-specific regulatory signatures, including both activating and repressive effects embedded within the enhancer architecture. These predictions are compared with CUT&RUN H3K27ac measurements following CEBPB knockout in a closely related myeloma model (RPMI 8226), enabling biological corroboration of directional effects across perturbation modalities. Despite differences between cell lines and perturbation conditions, several motifs exhibit consistent behavior, whereas others display context-dependent effects shaped by chromatin cofactors such as HDAC1. Together, these analyses demonstrate how sequence-level virtual perturbation can resolve motif-specific regulatory logic that complements and extends insights from experimental TF depletion. More broadly, the AlphaGenome-based framework offers a scalable approach for motif-level functional assessment and cis-regulatory analysis directly from DNA sequence, and can be extended to applications such as variant effect prediction and enhancer interpretation by prioritizing disease-associated noncoding variants that overlap nominated motif instances and quantifying their predicted regulatory impact via motif-instance ablation.

## Methods

### Overview of the analysis pipeline

**Figure 1:**
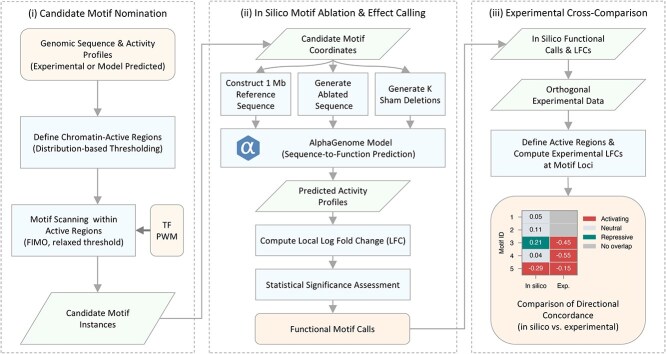
Overview of the AlphaGenome-based *in silico* motif-function detection workflow, which consists of three stages: (i) relaxed motif nomination within chromatin-active regions, (ii) motif-specific sequence ablation and effect calling using AlphaGenome predictions together with matched sham deletions to derive local LFC estimates, and (iii) cross-comparison with biological experimental perturbations to assess directional concordance.

We develop a TF-specific-ChIP-seq-independent framework for functional motif detection that integrates three stages: (i) motif nomination, (ii) AlphaGenome-based *in silico* motif ablation and effect calling, and (iii) biological experimental validation. Figure 1 summarizes the full analysis pipeline. In the first stage, candidate motif instances are identified within chromatin-active regions that are defined using either model-predicted or experimentally derived activity signals. Motif scanning is performed with Fimo [23] under deliberately relaxed significance thresholds so that weak, low-affinity, or context-dependent matches are retained. In the second stage, each nominated motif is subjected to *in silico* ablation: the motif sequence is precisely excised within AlphaGenome’s 1 Mb input window to generate an edited sequence, and the model is queried on both the original and ablated inputs. Localized differences in predicted chromatin activity provide an estimate of the motif’s functional contribution. In the third stage, motif-level predictions can be compared with biological experimental perturbations, including but not limited to TF depletion, reporter assays, CRISPR base editing, or allele-specific perturbations, allowing assessment of directional consistency between computational predictions and biological measurements without requiring TF-specific ChIP-seq.

In this study, we illustrate the method using H3K27ac as the regulatory readout and evaluate the predicted motif effects using matched TF–depletion experiments. The overall procedure is general and applies to any regulatory signal that AlphaGenome can predict for the relevant cell type.

### Step 1: Motif nomination

To restrict motif scanning to putative regulatory elements, chromatin-active regions are identified using a unified peak-calling strategy applied to chromatin activity profiles derived either from experimental assays (e.g. H3K27ac ChIP–seq or CUT&RUN) or from AlphaGenome-predicted signals. Because AlphaGenome outputs continuous activity values rather than read-based enrichment profiles, and CUT&RUN often lacks matched controls, conventional count-based peak callers such as MACS2 [24] are not uniformly applicable. We therefore adopt a distribution-based thresholding framework compatible with both experimental and model-derived signals.

Each chromosome is streamed once, and reservoir sampling is used to obtain an unbiased subsample of activity values. A genome-wide threshold is estimated from this pooled subsample; positions exceeding it are labeled active, and contiguous active bases are merged into intervals subject to a minimum-length filter. This yields a consistent set of candidate regulatory regions without assumptions tied to sequencing depth or background models.

Within each candidate interval, motif scanning is performed on the underlying DNA using Fimo (MEME Suite) with PWMs from databases such as JASPAR or CIS–BP. To maximize recall and retain weak or context-dependent sites, we use a relaxed significance threshold of $P \le 1 \times 10^{-3}$, less stringent than typical peak-centric cutoffs (e.g. $1 \times 10^{-4}$). All detected matches are retained for downstream *in silico* ablation and functional assessment.

### Step 2: AlphaGenome-based *in silico* motif ablation and effect calling

For each nominated motif $m$, a reference sequence is constructed by extracting a genomic window of length 1 Mb centered on the midpoint of its coordinates $(i_{m}, j_{m})$: 


\begin{align*} & S_{\mathrm{ref}}^{(m)} \in \{A,C,G,T\}^{1\,\mathrm{Mb}}. \end{align*}

This ensures that the motif and its surrounding cis-regulatory context fall within the receptive field of AlphaGenome.

To generate an ablated sequence, the nucleotides corresponding to the motif are removed from $S_{\mathrm{ref}}^{(m)}$, the two flanking segments are concatenated, and the sequence is padded from the distal window boundary with the corresponding downstream genomic bases to restore the full 1 Mb input length. AlphaGenome is then applied in track-prediction mode to both the reference and ablated sequences. For each input, AlphaGenome generates a predicted regulatory activity profile for the user-specified track.

To quantify the regulatory consequence of motif removal, the predicted signals are compared within a narrow local window $U$ anchored to the motif coordinates. A log_2_ fold change (LFC) is computed as 


\begin{align*} & \mathrm{LFC}^{(m)} = \log_{2}\!\left( \frac{\epsilon + \sum_{x\in U} H_{\mathrm{abl}}(x)} {\epsilon + \sum_{x\in U} H_{\mathrm{ref}}(x)} \right), \end{align*}

where $H_{\mathrm{ref}}(x)$ and $H_{\mathrm{abl}}(x)$ denote the predicted signal at position $x$ for the reference and ablated sequences, and $\epsilon $ is a small constant introduced to avoid numerical instability. The window $U$ is chosen to be much smaller than the surrounding 1 Mb context, reflecting the fact that TF motifs exert highly localized influences on regulatory activity.

To determine whether a candidate site exhibits a statistically significant perturbation, the observed LFC is compared with an empirical null distribution generated from length-matched sham deletions. For a given motif $m$ and its associated 1 Mb reference window, a set of $K$ sham ablations is constructed by selecting $K$ random control positions that do not overlap the motif coordinates $(i_{m}, j_{m})$, excising segments of the same length as the motif at those positions, and building ablated sequences in the same manner as described above. For each sham ablation, AlphaGenome is queried on the ablated sequence, and a sham LFC is computed relative to the fixed reference profile, yielding a collection of $K$ null LFC values. This background distribution captures the spatial heterogeneity in model predictions under length-matched deletions. A motif is designated as functional if its $\mathrm{LFC}^{(m)}$ lies outside the two-sided 95th percentile interval (2.5th to 97.5th percentiles) of this null distribution.

The direction of deviation provides a functional interpretation. LFC values below the lower percentile indicate that motif removal decreases predicted activity, consistent with an *activating* role in the examined chromatin context, whereas LFC values above the upper percentile indicate that deletion increases activity, consistent with a *repressive* influence. Motifs whose LFC values fall within the percentile interval are treated as *neutral*, indicating no detectable effect beyond background variability.

### Step 3: Biological experimental validation

Computationally inferred motif effects can be evaluated using orthogonal perturbations, including TF knockout or knockdown, CRISPR editing, reporter assays, or allele-specific perturbations. Because such experiments often alter broader regulatory programs rather than a single motif instance, we compare effect direction and magnitude as directional concordance rather than motif-isolated equivalence. In this study, this strategy is instantiated using CUT&RUN H3K27ac data following CEBPB knockout in multiple myeloma cells.

## Results

### Case study design and biological context

To assess the feasibility of the proposed computational framework in a biologically grounded setting, we designed a case study informed by prior perturbation experiments in RPMI 8226 multiple myeloma cells [25]. In that work, CRISPR–Cas9 knockout of CEBPB produced reproducible reductions in H3K27ac at a subset of dexamethasone-induced enhancers, with the upstream enhancer of *TSC22D3* (GILZ) showing one of the clearest examples of C/EBP$\mathrm{\beta} $-dependent regulation at both enhancer and gene-expression levels. This locus therefore provides a biologically validated test case for evaluating whether *in silico* motif ablation can recover motif-dependent regulatory signatures.

Because AlphaGenome currently supports only a limited set of hematopoietic contexts and does not include RPMI 8226, we performed *in silico* ablation in MM.1S, a closely related myeloma cell line with a similar enhancer landscape (global regulome profiling has reported consistent chromatin-accessibility patterns between MM.1S and RPMI 8226 [26]) and well-characterized glucocorticoid responsiveness. For MM.1S, AlphaGenome provides histone mark and chromatin accessibility predictions but does not include TF (ChIP–TF) outputs. Consequently, MM.1S TF-binding data—including C/EBP$\mathrm{\beta} $—were not part of the model’s training dataset, ensuring that the motif-level evaluation presented here is independent of the training process. Candidate C/EBP$\mathrm{\beta} $ motifs were nominated from MM.1S H3K27ac peaks and evaluated by *in silico* ablation within the MM.1S context.

For comparison with the knockout data, we focused on motif-containing loci active in both MM.1S and RPMI 8226, ensuring that concordance or discordance reflects CEBPB dependence rather than cell-line-specific chromatin differences.

Overall, this design anchors the computational analysis to an experimentally examined regulatory locus while accommodating the practical constraints of the AlphaGenome framework. The *TSC22D3* enhancer thus serves as a focused and biologically relevant case study for testing whether sequence-level virtual perturbation can identify functional C/EBP$\mathrm{\beta} $ motifs consistent with experimental chromatin responses.

### Motif nomination

Peak detection was first performed on H3K27ac profiles from MM.1S cells. A genome-wide activity threshold was estimated using reservoir sampling (sampling rate 0.01) applied across chromosomes 1–22, X, and Y, and the 75th percentile of the pooled sample was used as the activity cutoff. Within chr X, genomic positions exceeding this threshold were merged into continuous peak intervals, subject to a minimum length of 500 bp. These peak-defined regions delineate putative enhancers and provide the search space for downstream motif discovery.

To nominate C/EBP$\mathrm{\beta} $ motifs for subsequent functional analysis, a 60 kb window on chr X surrounding the *TSC22D3* locus (chrX:107 670 000–107 730 000) was selected. This interval encompasses the *TSC22D3* promoter together with the surrounding H3K27ac-defined enhancer cluster implicated by the experimental perturbation data, thereby capturing the full regulatory neighborhood relevant to C/EBP$\mathrm{\beta} $ function while, as a practical benefit, limiting the number of candidate motifs and the associated computational burden. Motif scanning was then performed using Fimo [23] and the CEBPB PWM from JASPAR (MA0466.3). A relaxed threshold ($P \le 1\times 10^{-3}$) was adopted to ensure high recall of candidate sites, as conventional Fimo cutoffs ($P \le 1\times 10^{-4}$) often miss weak but potentially functional motifs.

**Figure 2:**
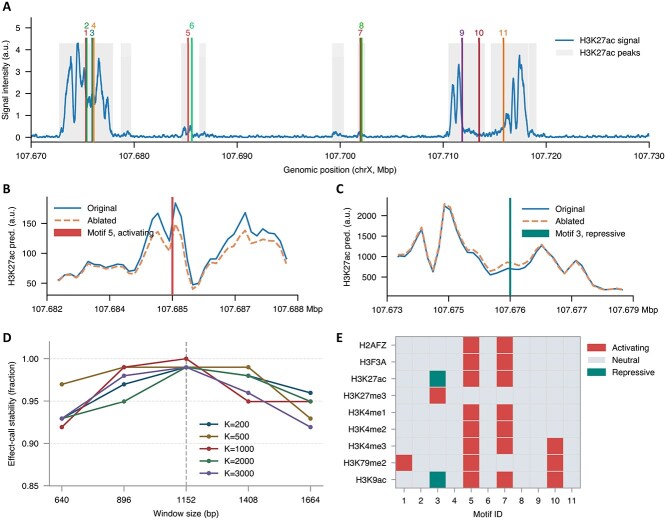
Integrated results of motif nomination, *in silico* ablation, and functional effect inference: (A) H3K27ac ChIP-seq signal in MM.1S cells (GEO: GSM894083), with shaded regions indicating called peak intervals and color bars marking the genomic positions of candidate C/EBP$\mathrm{\beta} $ motifs indexed from left to right with closely spaced motifs potentially overlapping due to limited plot resolution; (B–C) AlphaGenome-predicted H3K27ac regulatory activity profiles for reference and ablated sequences, showing an activating motif (ID 5) in (B) and a repressive motif (ID 3) in (C), with the ablated motif region marked by a vertical colored bar; (D) stability of effect calls across different local aggregation window sizes and null-distribution sampling depths; and (E) cross-mark summary of predicted activating, repressive, or neutral roles across nine histone modifications.

In total, 11 candidate motifs were detected within the H3K27ac-defined peak regions. Table 1 summarizes the genomic positions and FIMO-derived statistics of all candidate motifs, and Fig. 2A visualizes their placement within the H3K27ac landscape. Notably, 10 of the 11 candidates exhibited $P$-values greater than $1\times 10^{-4}$, indicating that standard FIMO settings would have excluded most sites.

**Table 1:** Candidate C/EBP$\mathrm{\beta} $ motifs detected by FIMO in MM.1S cells and their functional effects inferred from *in silico* motif ablation, including motif genomic coordinates, FIMO $P$-values, and the LFC in predicted H3K27ac signal resulting from deleting each motif, and functional roles indicate whether the observed LFC falls outside the two-sided 95% confidence interval derived from sham-deletion null distributions

**Motif ID**	**Start (bp)**	**End (bp)**	**FIMO $P$-value**	**LFC**	**Confidence interval**	**Functional role**	**Sham $P$-value**
1	107 675 323	107 675 334	$9.21\times 10^{-4}$	0.047	(−0.093, 0.199)	neutral	0.265
2	107 675 384	107 675 395	$4.24\times 10^{-4}$	0.109	(−0.074, 0.185)	neutral	0.114
3	107 675 922	107 675 933	$1.85\times 10^{-4}$	**0.214**	**(−0.098, 0.191)**	**repressive**	**0.046**
4	107 676 070	107 676 081	$1.06\times 10^{-4}$	0.043	(−0.122, 0.177)	neutral	0.277
5	107 685 228	107 685 239	$7.66\times 10^{-4}$	**−0.286**	**(−0.110, 0.152)**	**activating**	**0.014**
6	107 685 588	107 685 599	$4.99\times 10^{-5}$	0.172	(−0.085, 0.180)	neutral	0.056
7	107 701 953	107 701 964	$7.08\times 10^{-4}$	**−0.266**	**(−0.096, 0.163)**	**activating**	**0.016**
8	107 702 073	107 702 084	$8.78\times 10^{-4}$	−0.002	(−0.089, 0.195)	neutral	0.769
9	107 711 828	107 711 839	$2.34\times 10^{-4}$	0.046	(−0.090, 0.165)	neutral	0.269
10	107 713 472	107 713 483	$4.85\times 10^{-4}$	−0.096	(−0.112, 0.142)	neutral	0.055
11	107 715 841	107 715 852	$4.91\times 10^{-4}$	0.040	(−0.094, 0.161)	neutral	0.295

### *In silico* motif-effect inference across H3K27ac and additional chromatin activity profiles

Using the empirically selected hyperparameters (number of sham deletions $K=3000$ and evaluation-window width $U=1152$; see Supplementary), we quantified the regulatory contribution of each candidate C/EBP$\mathrm{\beta} $ motif within the *TSC22D3* locus. For each motif, the predicted change in chromatin activity following *in silico* ablation was summarized by an LFC within the local window and assessed for statistical significance using the sham-derived null distribution. A motif was designated as functional if its LFC lay outside the two-sided 95% confidence interval. We first examined H3K27ac, matching the experimental readout in RPMI 8226. Among the 11 candidate motifs, two exhibited activating effects (IDs 5 and 7), one showed a repressive effect (ID 3), and the remaining eight were classified as neutral. Representative activating (motif 5) and repressive (motif 3) examples are shown in Fig. 2B,C, with full results reported in Table 1 and Supplementary Fig. S4. Figure 2D provides a summary of the window-size stability pattern; full analyses appear in the Supplementary.

Because AlphaGenome simultaneously predicts nine histone modifications in MM.1S, we extended the ablation analysis to all nine histone tracks to obtain a broader view of how individual motifs influence their local regulatory environment. The multi-profile comparison, summarized in Fig. 2E, revealed two notable patterns. First, motifs with strong predicted effects on H3K27ac (motifs 3, 5, and 7) often showed coordinated perturbations across several additional histone modifications, indicating that their regulatory influence is not confined to a single modality. Second, some motifs (IDs 1 and 10) that appeared neutral with respect to H3K27ac nevertheless displayed detectable effects on other chromatin activity profiles, suggesting that H3K27ac alone may not capture the full functional spectrum of certain sites. To demonstrate the generalizability of our proposed method beyond a single locus-level example, we performed an independent supplementary case study in a PPARG-associated 60 kb window in human mesenchymal stem cells undergoing adipogenic differentiation [27, 28], integrating CEBPB ChIP-exo, H3K27ac, and AlphaGenome-based perturbation. Consistent with the interpretation above, the supplementary analysis showed that while only one high-confidence motif displayed a clear H3K27ac effect, all five ChIP-exo-supported motifs exhibited broader effects when all predicted histone marks were considered together (see Section 2.1 of Supplementary Material).

### Comparison with experimental CEBPB knockout

To evaluate whether AlphaGenome-based motif ablation captures bona fide C/EBP$\mathrm{\beta} $ dependence, we compared *in silico* motif-effect calls with CUT&RUN H3K27ac measurements obtained from C/EBP$\mathrm{\beta} $-deficient multiple myeloma cells, publicly available at the Gene Expression Omnibus (GEO, accession # GSE312300). CEBPB knockouts were generated in RPMI 8226 cells using CRISPR–Cas9 ribonucleoprotein (RNP)–mediated genome editing. Editing efficiency was assessed 48 hours after electroporation by PCR amplification of the targeted locus, Sanger sequencing, and indel profiling. Monoclonal knockout populations were subsequently derived by single-cell deposition, and loss of C/EBP$\mathrm{\beta} $ protein expression was confirmed by western blotting. Full experimental details are provided in [25].

For chromatin profiling, CUT&RUN H3K27ac was performed under two treatment conditions: dimethyl sulfoxide (DMSO), serving as the untreated baseline, and dexamethasone (DEX), a glucocorticoid receptor agonist that strongly induces transcription of *TSC22D3* [29]. We include the DEX arm to provide a treatment-shifted chromatin context in which enhancer acetylation is elevated at the locus, thereby making additional candidate motifs experimentally measurable. Concordant effects across DMSO and DEX provide stronger support for a motif-intrinsic contribution, whereas divergence is consistent with treatment-associated secondary remodeling effects beyond sequence-only perturbation. Each condition included three biological replicates of scramble controls and CEBPB knockouts [25]. Because AlphaGenome currently supports MM.1S but not RPMI 8226, predictions generated in MM.1S were used as a practical surrogate for approximating the chromatin activity landscape in RPMI 8226.

To determine which motifs reside in regions with sufficient chromatin activity to yield interpretable experimental responses, peak intervals were delineated from the RPMI 8226 CUT&RUN H3K27ac signal. For each treatment condition, genome-wide mean profiles were computed across control replicates, and peaks were identified using the activity-thresholding strategy described for MM.1S (reservoir sampling, 75th-percentile cutoff, minimum 500 bp length). These condition-specific intervals were intersected with the 11 candidate C/EBP$\mathrm{\beta} $ motifs nominated from MM.1S. Under DMSO, five of the 11 motifs overlapped RPMI 8226 peaks, whereas under DEX all 11 motifs fell within DEX-induced enhancer intervals surrounding the *TSC22D3* locus.

**Figure 3:**
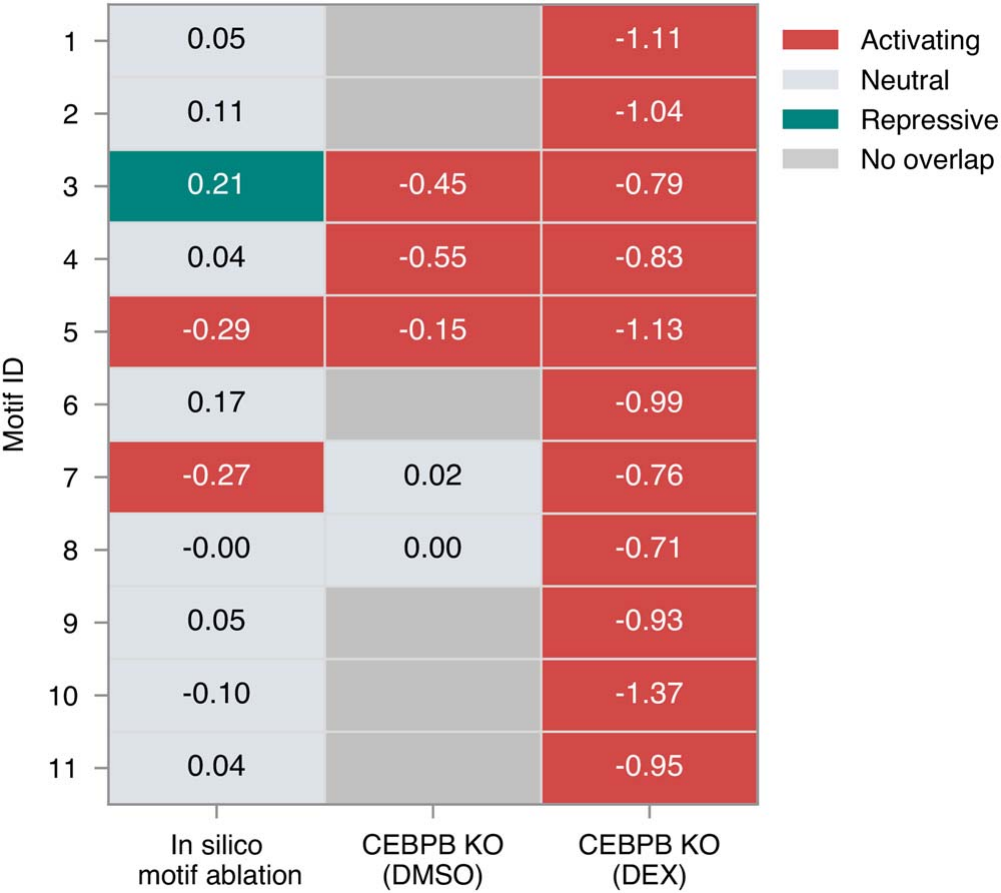
Heatmap of motif-level effect calls and LFC values across *in silico* and experimental conditions, with the first column showing predicted LFC and inferred motif effects from AlphaGenome-based ablation in MM.1S, the second and third columns showing CUT&RUN H3K27ac responses in RPMI 8226 under CEBPB knockout (KO) for DMSO and DEX, and “No overlap” indicating motifs outside the corresponding RPMI 8226 H3K27ac peak intervals and therefore excluded from experimental effect calling.

Figure 3 summarizes the *in silico* predictions and experimental chromatin responses across the three conditions. In DMSO-treated cells, five motifs reside within active peaks and therefore yield interpretable measurements. Among these, motifs 5 and 8 show concordant behavior: motif 5 exhibits an activating effect in both settings, meaning that CEBPB knockout reduces H3K27ac (negative LFC) at this locus, whereas motif 8 remains neutral. Motifs 4 and 7 show a shift in effect direction, with motif 4 transitioning from neutral *in silico* to activating experimentally and motif 7 transitioning from activating *in silico* to neutral. Motif 3 shows an opposite direction of effect, appearing repressive *in silico* but activating experimentally. Other motifs lie outside the RPMI 8226 H3K27ac peaks under DMSO and were therefore excluded from experimental effect calling. Their lack of an experimental readout does not contradict the model’s predictions but instead reflects insufficient chromatin signal at those sites.

Under DEX treatment, all 11 motifs fall within DEX-induced H3K27ac peaks and therefore provide measurable responses. In this condition, every motif exhibits an activating effect. Two motifs (5 and 7) match the *in silico* predictions, whereas motif 3 again reverses direction, switching from a repressive *in silico* effect to an activating experimental response. The remaining motifs transition from neutral *in silico* to activating under DEX. Overall, the experimental data reveal a broadly activating response under DEX, contrasting with the more heterogeneous effect distribution predicted *in silico*.

Taken together, these results highlight both the concordances and discrepancies between sequence-context ablation and experimentally measured chromatin activity. Notably, motif 3 consistently reverses effect direction, suggesting regulatory influences not captured by sequence-only perturbation; potential explanations are discussed in the Discussion section.

## Discussion

### Perturbation strategy and biological realism

We retain deletion as the primary operator for three reasons. First, AlphaGenome is fundamentally designed for direct sequence-editing comparisons, so localized insertions and deletions are natural within its prediction framework. Second, for cis-regulatory sequence analysis, deletion or excision provides a classical and direct way to test sequence necessity [30, 31]. Third, in our setting, deletion produced the clearest and most statistically robust signal and integrates naturally with a matched sham-deletion null distribution.

Alternative operators, such as dinucleotide-preserving shuffling [32], motif-to-neutral substitutions [30], reverse-complement inversions, positional shifts, and multi-copy motif insertions [31], provide more biologically nuanced perturbations because they preserve local sequence composition or higher-order syntax. To assess operator sensitivity, we additionally evaluated two conservative alternatives, dinucleotide-preserving shuffling and motif-to-neutral substitution, in our setting (see Supplementary Fig. S2). Consistent with their milder sequence changes, motifs with effects under these alternatives were contained within the set identified by deletion. We therefore report these alternatives as sensitivity analyses rather than exact biological surrogates. Additional discussion of AlphaGenome as the sequence-to-function model is provided in Supplementary Material S1.

For large receptive-field models such as AlphaGenome, deletion nevertheless provides a favorable balance between perturbation strength and statistical robustness. See Supplementary Material S1 for a justification of the use of AlphaGenome as our sequence-to-function oracle. Deep regulatory models including Basenji and Enformer are highly sensitive to localized edits [11, 12], and complete removal of the motif avoids artifacts that arise when partial motif information remains under mutation-based or shuffle-based strategies. Deletion also simplifies the estimation of null LFC values because length-matched sham deletions can be generated consistently across genomic regions.

### Choice of H3K27ac as the primary functional readout

H3K27ac is a well-established proxy for enhancer activation and correlates strongly with transcriptional output [14, 33]. Among histone modifications, it exhibits high signal-to-noise ratios in CUT&RUN and ChIP-seq measurements and is readily predicted by AlphaGenome’s high-resolution chromatin-activity heads. Using H3K27ac as the primary readout therefore provides a direct and interpretable measure of the regulatory consequences of motif perturbation, reflecting the combined influence of TF binding and local chromatin context. On the other hand, the same framework naturally extends to other chromatin modalities whenever matched experimental measurements are available.

Importantly, the absence of a detectable decrease in H3K27ac does not imply the absence of TF activity. Redundancy among nearby motif instances, cooperative binding with other factors, or chromatin buffering mechanisms may preserve enhancer activation even when a single motif is disrupted. To guard against overinterpreting such subtle fluctuations, we evaluate motif effects relative to empirical null distributions derived from length-matched sham deletions, which provide a principled baseline for assessing statistical significance.

### Contextualizing model–experiment discrepancies in chromatin regulation

Comparison between *in silico* motif ablation and experimental CEBPB knockout revealed a key inconsistency at motif 3 under DMSO: AlphaGenome predicted a repressive role, whereas CUT&RUN in RPMI 8226 cells indicated an activating effect. *In silico* deletion of motif 3 increased predicted H3K27ac, consistent with loss of a repressive element, while CEBPB knockout decreased H3K27ac at the same locus.

**Figure 4:**
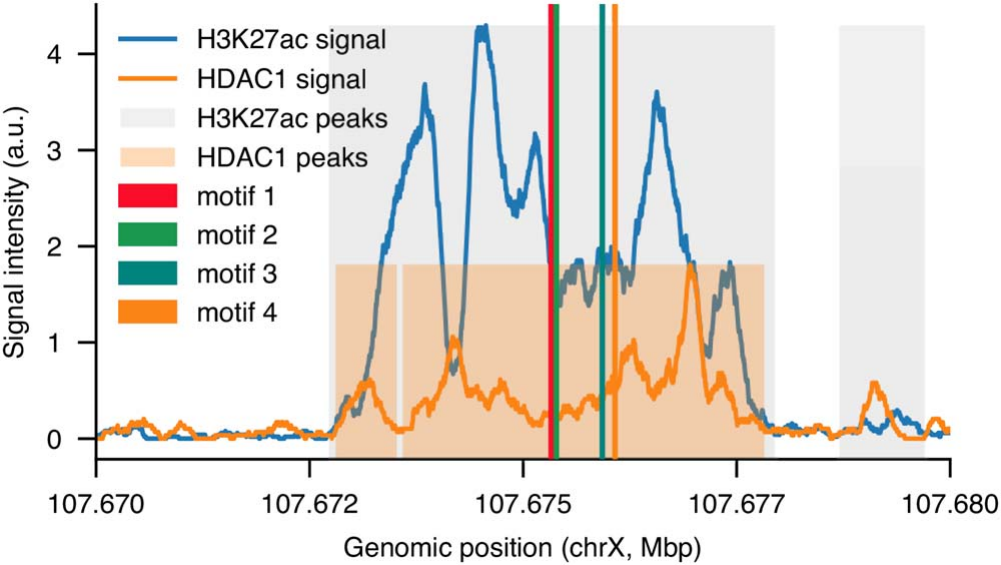
ChIP–seq profiles of H3K27ac and HDAC1 in MM.1S cells (GEO: GSM894083 and GSM2302869, respectively) across the genomic interval containing motifs 1–4.

This discrepancy is biologically interpretable. Figure 4 shows that motif 3 lies within a strong HDAC1 peak in MM.1S cells, and a broader 60 kb view (Supplementary Fig. S5) indicates that nine of eleven candidate motifs fall in regions co-enriched for H3K27ac and HDAC1. Although motifs 1, 2, and 4 also reside in this HDAC1-enriched subregion, motifs 1 and 2 fall outside the RPMI 8226 H3K27ac peaks under DMSO and thus lack experimental effect calls, whereas motif 4 is addressed separately below.

HDAC1 interacts with C/EBP$\mathrm{\beta} $ complexes and can act as a local corepressor that reduces acetylation [34, 35]. Deleting motif 3 computationally isolates its contribution to this putative repressive complex, yielding a localized increase in predicted acetylation. By contrast, CEBPB knockout removes all CEBPB-dependent activating inputs across the enhancer, producing a locus-wide decrease in H3K27ac that masks the subtle local effect expected from loss of a single repressive motif.

Several other motifs show treatment- or context-dependent shifts between *in silico* and experimental calls. Under DMSO, motif 4 appears neutral *in silico* but activating experimentally, whereas motif 7 shows the opposite pattern. Under DEX, however, all motifs exhibit activating effects. DEX strongly activates the *TSC22D3* locus, causing widespread H3K27ac gain and extensive cofactor recruitment; CEBPB knockout therefore primarily reflects loss of global activating input rather than motif-specific perturbations. Thus, even motifs predicted neutral or repressive *in silico* appear activating experimentally because the dominant effect is DEX-induced activation being removed. These transitions illustrate that *in silico* ablation isolates intrinsic motif contributions, whereas TF knockout captures aggregate consequences of factor loss in a dynamically remodeled chromatin environment.

More broadly, discrepancies reflect the fundamentally different perturbation modalities. AlphaGenome ablation probes motif-specific logic within an intact chromatin background, whereas CRISPR-mediated knockout removes the TF globally, inducing secondary effects including loss of activating inputs, altered cofactor recruitment, and potential chromatin restructuring [36]. Baseline differences between MM.1S and RPMI 8226 in chromatin state or cofactor availability may further shape locus-specific responses.

These considerations motivate a principled interpretation. Our comparison is intended as an assessment of directional concordance rather than exact equivalence between the two perturbation modalities. Concordant sites are especially informative because they provide convergent evidence from two independent directions and therefore represent high-confidence functional motifs. By contrast, divergent cases should not be viewed as false positives. Rather, they highlight situations in which motif-intrinsic effects may be obscured by broader cellular or chromatin-level responses in bulk knockout assays. Motifs with concordant directionalities, such as motifs 5 and 8 under DMSO or motifs 5 and 7 under DEX, therefore represent the strongest candidates for targeted follow-up perturbations such as CRISPR base editing, allele-specific disruption, or reporter assays.

Overall, these findings underscore the complementary strengths of *in silico* and experimental perturbation: computational ablation provides scalable, motif-resolved insight directly from DNA sequence, whereas TF knockout captures integrated chromatin responses at the system level. Together, these approaches yield a more complete mechanistic understanding of enhancer regulation.

### Methodological considerations and limitations

Although AlphaGenome-based virtual ablation offers a fast and interpretable way to evaluate motif function, several methodological caveats warrant discussion. First, AlphaGenome is a predictive model and may yield inaccurate signals in specific genomic contexts. In addition, because the experimental measurements and the model predictions are not obtained under fully matched conditions, detecting and attributing such model errors can be challenging in our setting.

Second, single-site perturbations measure only the marginal contribution of an individual motif. Enhancers often contain clusters of cooperating motifs with spacing- or orientation-dependent syntax [37], meaning that the effect of a single deletion may underestimate or misrepresent how a motif behaves within its native combinatorial context. Such cooperative grammar likely contributes to some *in silico* versus experimental discrepancies at the TSC22D3 locus.

Third, although the revised study now includes an additional independent supplementary case study at a PPARG-associated locus in human mesenchymal stem cells, the current validation scope remains limited. The main text still relies on a single matched perturbation setting, namely comparison between AlphaGenome-based motif ablation and CEBPB knockout with H3K27ac readout in multiple myeloma cells, whereas the supplementary PPARG analysis serves as orthogonal support rather than a fully matched perturbation experiment. Therefore, the present work should be interpreted as a protocol-focused demonstration and motif-prioritization framework rather than a systematic benchmark across diverse loci, TFs, perturbation types, and cellular contexts.

Future work should extend the framework to additional TFs, broader sets of loci, and more fully matched experimental perturbation settings, including locus-specific editing assays that can test nominated motif instances more directly in their native chromatin context.

## Conclusion

This study demonstrates that large multimodal sequence models such as AlphaGenome can enable scalable and informative motif-level functional inference directly from DNA sequence. By combining relaxed motif nomination with targeted *in silico* ablation, our framework identifies activating, repressive, and neutral motifs without requiring TF-specific ChIP–seq. Comparison with CEBPB knockout experiments highlights both concordant cases, where AlphaGenome-inferred motif effects are directionally consistent with experimental chromatin responses, and biologically interpretable discrepancies that arise when motif-specific logic is masked by global TF depletion or treatment-driven chromatin remodeling. These findings illustrate the complementary nature of computational and experimental perturbations and support sequence-only virtual ablation as a hypothesis-generation and motif-prioritization strategy for regulatory annotation. Broader validation across additional TFs, loci, perturbation types, cell types, and chromatin modalities will be necessary to establish its general applicability.

**Key points**

- We present a framework for functional motif inference that links relaxed candidate nomination with statistically calibrated effect calling through targeted *in silico* ablation using AlphaGenome.
- The framework recovers activating, repressive, and neutral motifs directly from DNA sequence without requiring TF-specific ChIP–seq, and provides high-confidence motif-level necessity estimates through a matched sham-deletion null distribution.
- Cross-cell-line comparison between MM.1S *in silico* predictions and RPMI 8226 CEBPB knockout experiments reveals both concordant motifs and biologically interpretable discrepancies, clarifying when motif-intrinsic logic is masked by global TF depletion or treatment-dependent chromatin remodeling.
- The proposed strategy offers a scalable, interpretable, and experimentally testable paradigm for cis-regulatory motif analysis, with potential applicability to additional transcription factors, chromatin modalities, and cellular contexts, subject to further validation.

## Supplementary Material

supplementary_bbag422

biographical_notes

## Data Availability

The source code and *in silico* analysis data are publicly available at https://github.com/liangyuxuan1/Motif-Detection. The experimental CEBPB knockout data are publicly available at Gene Expression Omnibus (GEO, accession # GSE312300).
