# Supplementary material for: Functional motif detection via in silico ablation using AlphaGenome
Source: Brief Bioinform. 2026 Aug 6;27(4):bbag422. doi: 10.1093/bib/bbag422 (PMC13446514; doi:10.1093/bib/bbag422)
Supplement: Supplementary file 1 [file supplementary_bbag422.pdf]

Supplementary Material for

Functional Motif Detection via In Silico Ablation Using AlphaGenome

Yuxuan Liang[1], Sebastian A. Dziadowicz[2], Lei Wang[2], Gangqing Hu[2,*], Pingkun Yan[1,*], and Ge Wang[1,*]

[1]Department of Biomedical Engineering, Rensselaer Polytechnic Institute, Troy, NY 12180, USA

[2]Department of Microbiology, Immunology and Cell Biology, West Virginia University, Morgantown, WV 26506-9177, USA

[*]Corresponding authors: michael.hu@hsc.wvu.edu (G. Hu), yanp2@rpi.edu (P. Yan), wangg6@rpi.edu (G. Wang)

1. Supplementary Discussion

1.1. Reliability of AlphaGenome as a sequence-to-function oracle

AlphaGenome provides a strong, general-purpose sequence-to-function predictor, as supported by its benchmark performance against prior long-context models (e.g., Enformer/Basenji2-style targets) across diverse regulatory readouts (Avsec et al., 2021; Kelley, 2020; Avsec et al., 2026). In particular, AlphaGenome supports megabase-scale input context and high-resolution outputs (up to 1 bp for certain modalities and 128 bp for most chromatin tracks), capturing both distal regulatory context and fine-scale perturbations within a unified framework (Avsec et al., 2026). The model was evaluated against the strongest available external baseline for each task rather than against every earlier sequence model individually. As a result, its comparisons covered multiple task-specific state-of-the-art models, including Borzoi, Enformer, and ChromBPNet, and the paper reported state-of-the-art performance on most evaluated genome-track and variant-effect benchmarks. A key motivation for the use of AlphaGenome in our study is for cell-type specificity. AlphaGenome is trained on a large collection of functional genomic tracks spanning many tissues and cell types, and it provides chromatin predictions for the MM.1S myeloma context (Avsec et al., 2026). In contrast, many widely used public sequence-to-function models expose fixed target sets and do not directly provide MM.1S-specific tracks. Thus, producing matched outputs of these models would require retraining or substituting a different cell type, which would confound a comparison in our locus-specific case study.

Finally, AlphaGenome is well-suited for motif-instance perturbation because it is designed to respond to small, localized sequence edits. Therefore, it is natural for us to use AlphaGenome for motif-instance ablation (paired with length-matched sham deletions) to quantify predicted regulatory consequences in a statistically calibrated manner, even in settings where matched locus-specific experimental chromatin labels are unavailable.

To further assess predictive fidelity in our setting, we performed an additional validation experiment in the MM.1S context. We randomly sampled ten 1 Mb genomic regions across the genome, used AlphaGenome to generate H3K27ac predictions for each region, and compared these predictions with the corresponding experimental measurements. This analysis yielded an average area under curve (AUC) of 0.733, supporting that AlphaGenome provides reasonably accurate H3K27ac track predictions in MM.1S for the type of large scale

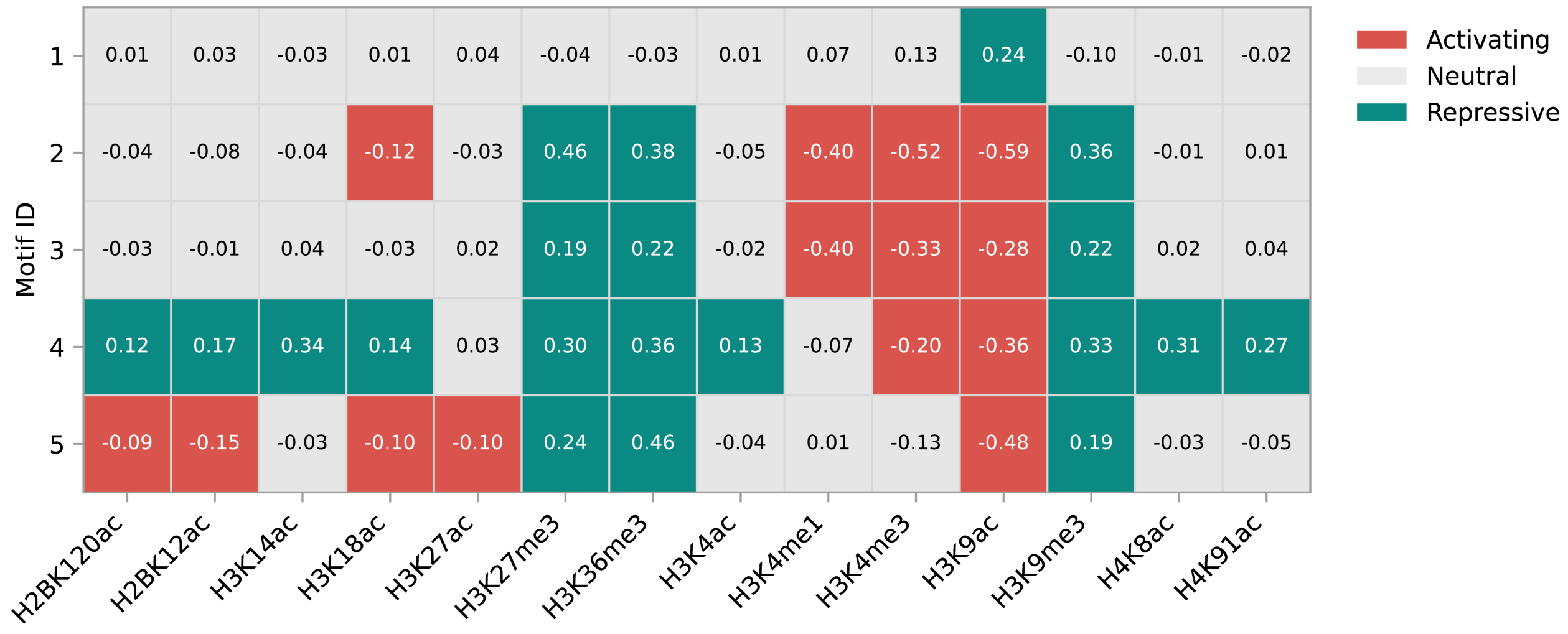

Supplementary Figure S1: Independent supplementary case study at a PPARG-associated locus in human mesenchymal stem cells undergoing adipogenic differentiation. Cross-mark summary across all available histone outputs.

genomic windows used in this study.

2. Supplementary Results

2.1. Independent supplementary case study at a PPARG-associated locus

To reduce reliance on a single locus-level example, we performed an additional supplementary case study in a PPARG-associated 60 kb window (hg19 chr3:12,386,733–12,446,733) in human mesenchymal stem cells undergoing adipogenic differentiation. This analysis integrated high-resolution CEBPB ChIP-exo data from GSE111515 Cohen et al. (2018) with H3K27ac data from GSE68864 Cohen et al. (2015).

Within this window, 10 CEBPB motif instances were supported by the ChIP-exo-derived motif annotations. We also performed H3K27ac-guided motif nomination within the same region. Because these two nomination routes did not fully overlap, we rescanned the entire 60 kb window with FIMO using the CEBPB motif MA0466.3 ($p \leq 10^{-3}$). This whole-window scan yielded 107 hits, among which only five overlapped directly with the ChIP-exo-supported motifs. We therefore treated these five sites as a high-confidence core motif set for downstream analysis.

AlphaGenome-based perturbation of these five high-confidence motifs showed that only one motif (chr3:12435629–12435639) displayed a clear activating effect on H3K27ac. At the same time, all five motifs showed a non-neutral effect in at least one of the broader set of predicted histone-mark outputs, indicating that these ChIP-exo-supported sites are not functionally inert even when their H3K27ac-specific effect is weak. This supplementary analysis therefore provides additional support that the proposed framework can refine a broad candidate pool into a smaller high-confidence motif set and reveal motif-associated regulatory effects beyond a single chromatin readout.

2.2. Sensitivity to perturbation operators beyond deletion

Our use of deletion as the primary in silico perturbation operator is not intended to imply exact mechanistic equivalence to the biological CEBPB knockout experiment in the main text. Rather, deletion is used here as

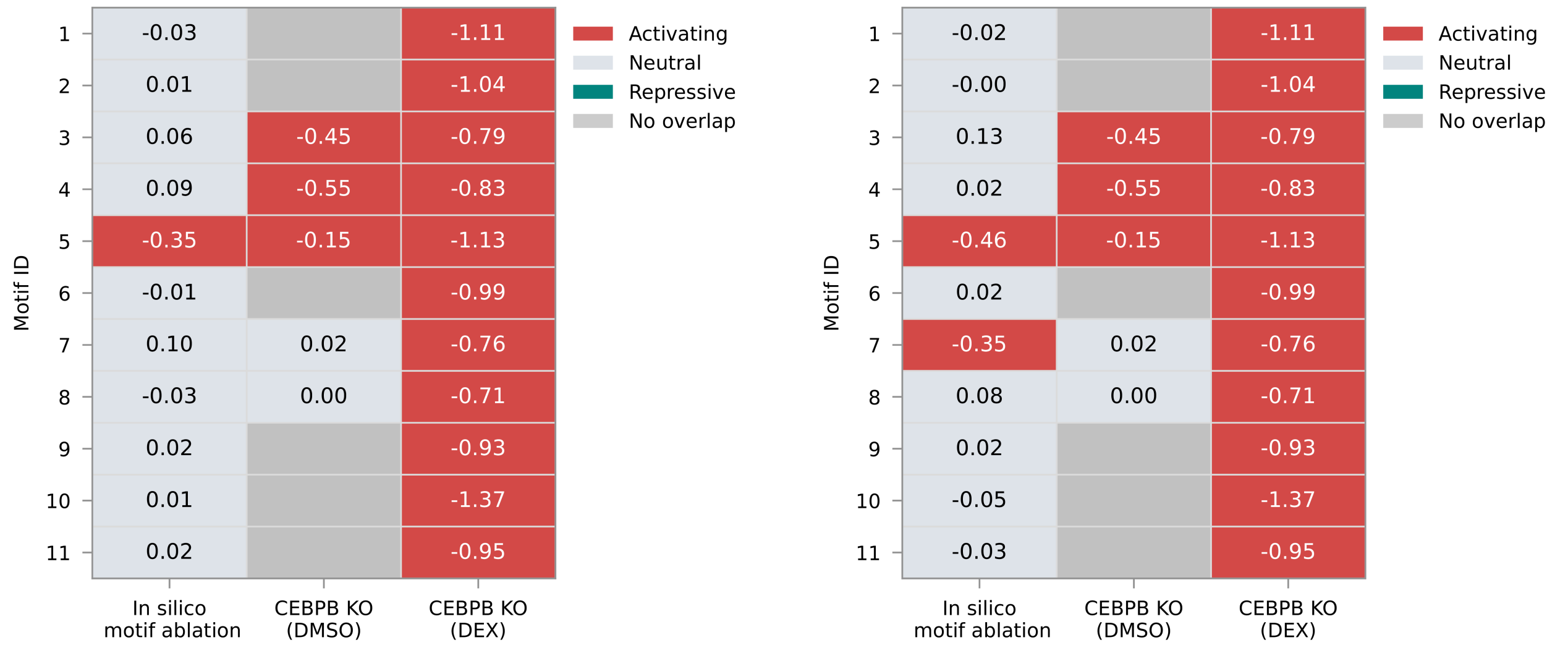

Supplementary Figure S2: Sensitivity analysis of perturbation operators beyond deletion. (a) dinuc_shuffle; (b) neutral_substitution.

a strong and well-defined sequence perturbation for testing motif necessity at the sequence level, whereas transcription factor knockout removes the factor globally and can induce broader secondary chromatin remodeling effects. We retain deletion as the primary operator because it produced the clearest and most statistically robust signal in our setting and integrates naturally with a matched sham-deletion null distribution.

To evaluate whether motif-level effect calling depends on the perturbation operator, we extended the framework beyond deletion and tested two alternative operators: (i) dinucleotide-preserving shuffle (`dinuc_shuffle`) and (ii) motif-to-neutral substitution (`neutral_substitution`).

Perturbation operators. For each motif instance, we compared:

Dinucleotide-preserving shuffle (`dinuc_shuffle`). Shuffle nucleotides within the motif interval while preserving dinucleotide counts, aiming to disrupt motif syntax while maintaining local sequence composition.

Motif-to-neutral substitution (`neutral_substitution`). Substitute the motif interval with a length-matched "neutral" sequence designed to reduce motif-like information while keeping basic sequence characteristics.

Results. Unlike deletion, both `dinuc_shuffle` and `neutral_substitution` preserve part of the local sequence characteristics (e.g., composition or short-range dependencies). In practice, the resulting perturbed sequence within the motif interval is often not as drastically altered as full removal, leading to smaller predicted changes in regulatory outputs. Across the evaluated motif instances, motif instances showing clear effects under `dinuc_shuffle` or `neutral_substitution` were contained within the set of motif instances identified as significant under deletion (Supplementary Figure S2). These results indicate that deletion provides a stronger and more sensitive loss-of-function perturbation signal in our setting, while the alternative operators serve as a robustness check consistent with the main conclusions.

2.3. Hyperparameter selection for motif ablation analysis

After candidate CEBPB motifs were nominated, their local regulatory effects were assessed by in silico motif ablation using AlphaGenome configured to output the `CHIP_HISTONE` track type. For each candidate, both the

reference 1 Mb context sequence and an ablated version in which the motif segment was deleted were submitted to AlphaGenome, which returns predicted H3K27ac profiles for each input. The difference between the reference and ablated prediction profiles within a local genomic neighborhood provides the basis for determining whether the motif exerts an activating, repressive, or neutral effect.

Motif-effect inference depends on two key hyperparameters: the number of sham deletions (K) used to construct the empirical null distribution and the width of the evaluation window (U) over which local signal changes are aggregated. Both parameters influence the stability and robustness of the resulting effect calls. Because the `CHIP_HISTONE` setting of AlphaGenome produces nine histone modification tracks for MM.1S, all nine tracks were included when evaluating hyperparameter sensitivity, thereby increasing the number of independent measurements and providing a more reliable assessment of how variations in K and U affect motif classification.

Choice of the number of sham deletions (K)

To determine an appropriate number of sham deletions, we systematically evaluated $K \in \{200, 500, 1000, 2000, 3000\}$ for each motif. Because larger K values provide a more stable empirical null distribution, the setting $K = 3000$ was treated as a high-confidence reference against which all smaller K values were compared. For each K, effect-call consistency was assessed by computing the proportion of {motif, histone track, U width} combinations whose functional labels differed from those obtained under the $K = 3000$ reference. A disagreement was counted whenever the effect label differed from the corresponding label under $K = 3000$. Relative to the $K = 3000$ baseline, settings of $K = 200$, 500, 1000, and 2000 produced 6.3%, 5.7%, 5.1%, and 5.1% discrepant calls, respectively. These results indicate that small K values introduce greater stochastic variability into the estimated null distribution, whereas values of K around 1000 or higher yield more stable and reproducible effect calls.

The statistical benefits of increasing K must, however, be weighed against the associated computational cost. As summarized in Supplementary Table 1, the total runtime grows superlinearly with K, rising from 44.5 minutes at $K = 200$ to more than 10 hours at $K = 3000$. Because the dominant contribution to runtime comes from repeated AlphaGenome requests, whose latency depends on both network conditions and the instantaneous load of the external inference service, the absolute runtimes should be viewed as approximate and system dependent. Even so, the relative trend is robust: while intermediate choices such as $K = 1000$ or 2000 reduce runtime, they still produce a measurable 5.1% disagreement relative to the $K = 3000$ reference. Balancing these considerations, and noting that total runtime scales primarily with the number of AlphaGenome queries rather than local computational resources, we adopted $K = 3000$ as a practical operating point, which provides the most stable and reliable null distribution among the tested configurations.

Supplementary Table 1: Runtime cost of in silico analysis for different number of sham deletions (K). Experiments ran on a workstation with an AMD EPYC 7502 CPU (32 cores, 64 threads) and 512 GB RAM, with AlphaGenome accessed via a 1 GbE campus network.

K	**AlphaGenome cost (min)**	**Total cost (min)**
200	42.3	44.5
500	104.8	107.0
1000	211.9	214.3
2000	413.9	417.0
3000	620.5	623.2

Choice of the local aggregation window (U)

The effect of local window width on motif-effect inference was next examined. Because AlphaGenome predicts chromatin activity in 128 bp bins, evaluation windows were constructed by combining the motif-containing bin with a symmetric number of neighboring bins. Five window widths (640–1664 bp) were considered, corresponding to the motif bin and its ± 2 to ± 6 neighbors. These values span biologically plausible regulatory neighborhoods: transcription factor binding typically influences chromatin structure over several hundred base pairs, whereas histone modifications such as H3K27ac extend across broader enhancer regions. The selected windows thus provide a controlled progression from highly localized to moderately extended contexts.

Robustness across window sizes was quantified using an effect-call stability metric. For each motif, a consensus effect was defined as the most frequent label (activation, repression, or neutral) across the five window widths; no ties occurred. Stability at a given window size was then measured as the fraction of {motif, histone mark} combinations whose effect matched the corresponding consensus, producing a value between 0 and 1 that reflects the consistency of motif-effect inference under perturbations to the evaluation window.

The resulting stability patterns are shown in Fig. 2D of the main text. Across all K values, stability exhibited a consistent trajectory: it increased from $U = 640$ bp to 1152 bp, achieved its maximum at $U = 1152$ bp (0.99–1.00 for every K), and declined at larger windows (1408 and 1664 bp). This reproducible pattern indicates that small windows can omit relevant contextual signal, whereas overly broad windows can dilute motif-specific perturbations by aggregating in unrelated regulatory activity.

Further insight is provided in Supplementary Fig. S3, which displays predicted LFC values across window sizes together with the corresponding sham-derived 95% confidence intervals at $K = 3000$. These profiles show that effect calls may shift when U is either too narrow or too large. For example, motif 3 falls within the confidence interval at $U = 640$ bp but lies outside it when the window is expanded, yielding a different functional interpretation. Such cases highlight the importance of selecting a window that balances contextual inclusion with localization.

Together, these observations identify $U = 1152$ bp as the most reliable operating point for motif-effect analyses in the main text.

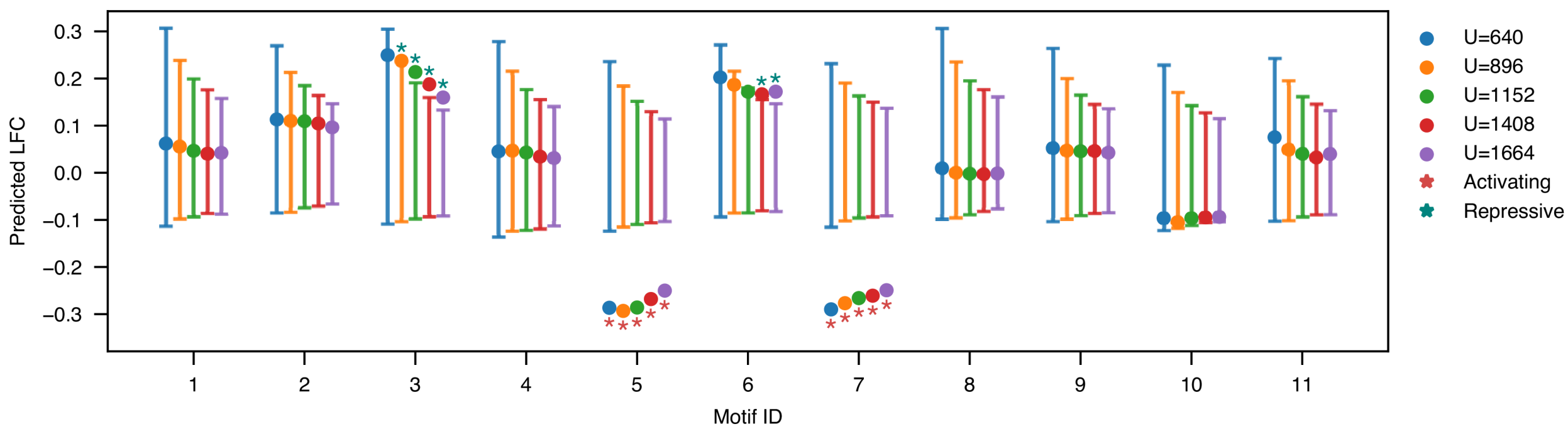

Supplementary Figure S3: Sensitivity of in silico motif-effect calls to the evaluation window size U in MM.1S H3K27ac predictions. For each (motif, U) pair, the colored point denotes the predicted LFC and the vertical error bar indicates the empirical 95% confidence interval obtained from $K = 3000$ matched sham deletions. The motif-effect classification is indicated by an overlaid symbol: red asterisks for activating calls, blue asterisks for repressive calls, while neutral calls are left unmarked.

3. Supplementary Figures

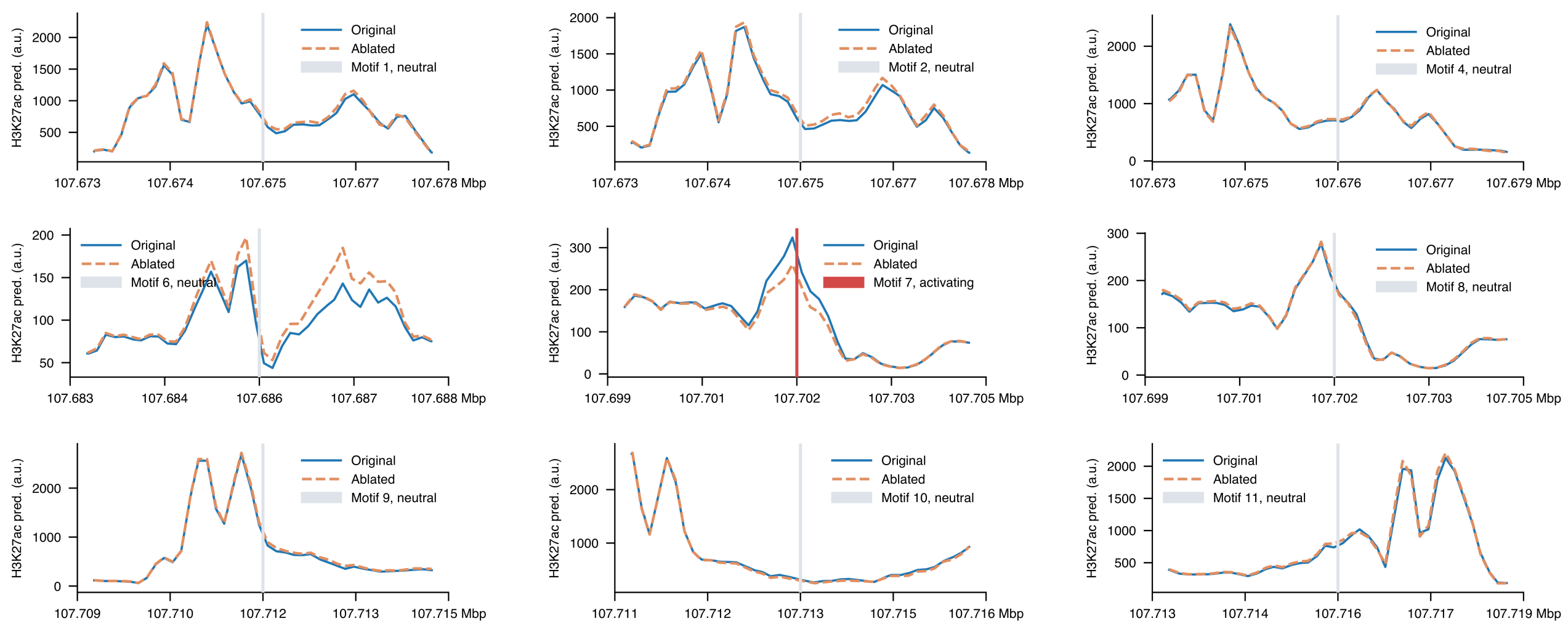

Supplementary Figure S4: AlphaGenome-predicted H3K27ac signals before and after motif ablation for nine CEBPB motif candidates. Each panel shows the original (solid) and ablated (dashed) tracks; the ablated motif segment is indicated by the vertical color bar.

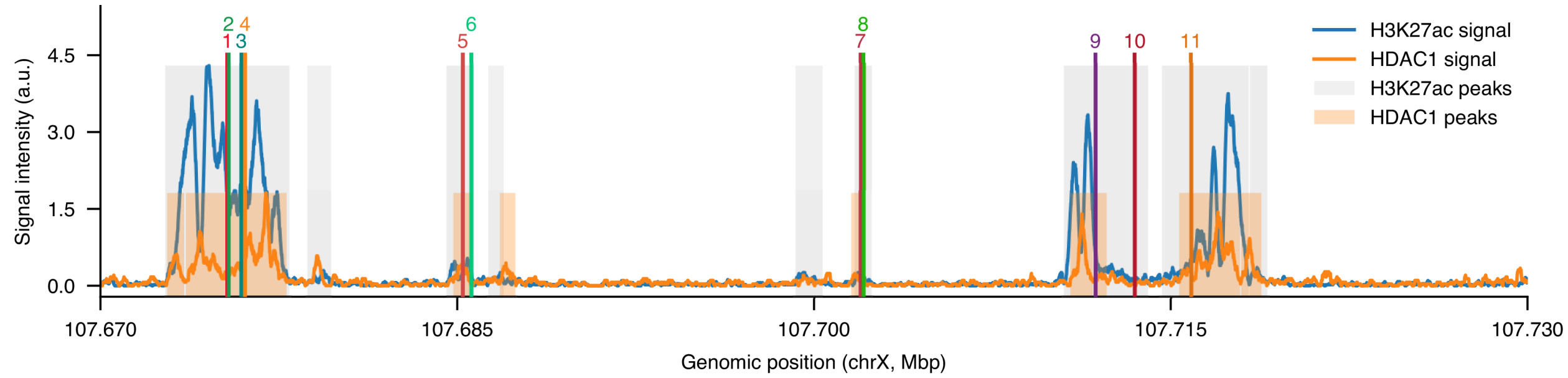

Supplementary Figure S5: H3K27ac and HDAC1 ChIP–seq profiles across the TSC22D3 locus in MM.1S cells. The blue and orange curves show H3K27ac and HDAC1 signal intensities, respectively. Shaded gray regions denote H3K27ac peaks, and shaded orange regions denote HDAC1 peaks. Vertical colored bars mark the genomic positions of the 11 candidate C/EBPβ motifs (indexed from left to right; closely spaced motifs may visually overlap due to limited plot resolution). Nine of the 11 motifs (all except motifs 6 and 10) lie within regions showing co-enrichment of H3K27ac and HDAC1 ChIP–seq signal, indicating that most candidate motifs reside in enhancer-associated chromatin that exhibits both acetylation and deacetylase occupancy.

References

Avsec Ž, Agarwal V, Visentin D. et al. Effective gene expression prediction from sequence by integrating long-range interactions. *Nature Methods*, 18(10):1196–1203, 2021. https://doi.org/10.1038/s41592-021-01252-x.

Kelley DR. Cross-species regulatory sequence activity prediction. *PLOS Computational Biology*, 16(7):1–27, 07 2020. https://doi.org/10.1371/journal.pcbi.1008050.

Avsec Ž, Latysheva N, Cheng J. et al. Advancing regulatory variant effect prediction with alphagenome. *Nature*, 649:1206–1218, 2026. https://doi.org/10.1038/s41586-025-10014-0.

Cohen Daniel M., Won K., Nguyen N.. et al. Shared nucleotide flanks confer transcriptional competency to bzip core motifs. *Nucleic Acids Research*, 46(16):8371–8384, 2018. https://doi.org/10.1093/nar/gky681.

Cohen Daniel M., Yang Ming, Tarumi Takaoki. et al. Atf4 licenses c/ebpβ activity in human mesenchymal stem cells primed for adipogenesis. *Elife*, 4:e06821, 2015. https://doi.org/10.7554/eLife.06821.
